# Influence of Different Closure Systems on Yeast Metabolism During the Secondary Fermentation of Traditional Method Sparkling Wines

**DOI:** 10.3390/microorganisms14081623

**Published:** 2026-07-24

**Authors:** Sara Sofia Pinheiro, Maria João Cabrita, Marco Gomes da Silva

**Affiliations:** 1 LAQV/REQUIMTE, Department of Chemistry, NOVA School of Science and Technology, NOVA University Lisbon, 2829-516 Caparica, Portugal; ss.pinheiro@campus.fct.unl.pt; 2 MED, Mediterranean Institute for Agriculture, Environment and Development & CHANGE, Global Change and Sustainability Institute, Departamento de Fitotecnia, Escola de Ciências e Tecnologia, Universidade de Évora, Polo da Mitra, Ap. 94, 7006-554 Évora, Portugal; mjbc@uevora.pt

**Keywords:** sparkling wines, secondary fermentation, *tirage* closures, amino acid evolution, volatile composition

## Abstract

Secondary fermentation is a critical stage in the production of traditional method sparkling wines, during which yeast metabolism drives major changes in wine composition. However, the biochemical processes occurring during this phase and the influence of bottle closure systems remain poorly understood. This study evaluated the evolution of basic oenological parameters, free amino acids and volatile compounds during the first 90 days of secondary fermentation under two bottle closure systems: crown cap and *tirage*-cork. Fermentation time was the principal factor governing wine composition, with the most pronounced metabolic changes occurring between 30 and 45 days after bottling. Active nitrogen metabolism was evidenced by substantial amino acid redistribution, particularly involving ASN, ORN, AAA, GLU and ASP, while the accumulation of higher alcohols and other fermentation-derived volatiles reflected intense yeast metabolic activity during bottle fermentation. Although both closure systems successfully completed fermentation, *tirage*-cork-fermented wines showed a tendency towards faster fermentation progression and distinct patterns of amino acid and volatile compound development. Pearson correlation analysis revealed generally weak to moderate associations between amino acids and their corresponding volatile compounds, supporting the view that aroma formation results from the integration of multiple metabolic processes rather than from precursor availability alone. Overall, the results suggest that closure-associated metabolic divergence becomes detectable during the active phase of secondary fermentation and may contribute to differences in nitrogenous and aroma-related compounds. These findings suggest that bottle closure may represent an important technological variable associated with differences in the metabolic and compositional trajectories observed during the active phase of secondary fermentation. Given the limited biological replication of the present study, these observations should be interpreted with caution and warrant confirmation through studies incorporating biological replication and direct measurements of oxygen transfer.

## 1. Introduction

Sparkling wines are special wines produced from grapes, musts, or base wines using techniques accepted by the OIV and are characterized upon uncorking by a more or less persistent effervescence resulting from the release of carbon dioxide of exclusively endogenous origin [1]. Among the different production methods, the traditional method is particularly associated with premium sparkling wines and involves a secondary fermentation carried out inside the bottle (known as *tirage* or *prise de mousse*), followed by a prolonged aging period on lees [2]. During aging, yeast (Throughout this manuscript, the term "yeast" is used in its conventional food microbiology context to refer to unicellular fungi involved in alcoholic fermentation, particularly *Saccharomyces cerevisiae*) cells undergo autolysis, leading to the release of intracellular compounds such as amino acids, peptides, polysaccharides, and lipids into the wine matrix [3,4]. These compounds are known to influence several quality attributes, including mouthfeel, foam stability, and aroma, contributing to the development of characteristic sensory notes such as toasted, brioche, and nutty descriptors [5]. As a result, the aging stage has been extensively studied, and its role in shaping the final quality of sparkling wines is well established [5,6,7,8,9].

In contrast, the secondary fermentation phase itself has received comparatively less attention. Often considered primarily as the stage responsible for carbon dioxide production, this phase is, in fact, a highly active biochemical period. During the so-called *prise de mousse*, yeast cells remain metabolically active under increasingly stressful conditions, including high ethanol content, low pH, nutrient limitation, and increasing internal pressure [2,5]. Under these conditions, yeasts actively consume sugars and nitrogen compounds while producing a wide range of secondary metabolites that can significantly influence the chemical composition of the wine [6]. This metabolic activity plays a key role in defining the initial aromatic profile and the pool of precursors available for subsequent aging. Among these compounds, amino acids are of particular relevance, as they constitute the main source of assimilable nitrogen for yeast metabolism and act as precursors for the formation of volatile compounds such as higher alcohols and esters [6,7,9,10]. In particular, amino acid catabolism through the Ehrlich pathway leads to the formation of higher alcohols via transamination, decarboxylation, and reduction reactions, linking nitrogen metabolism directly to aroma compound production [11]. Therefore, changes in amino acid composition during the secondary fermentation may have direct implications for aroma development. Although amino acid metabolism has been extensively investigated during primary alcoholic fermentation and, to a lesser extent, during prolonged lees aging, comparatively little is known about the temporal development of free amino acids during the active phase of secondary fermentation in the bottle, particularly in relation to different closure systems.

In addition to the intrinsic characteristics of the grape must and yeast strain, several technological factors throughout the production process can influence the chemical composition of the final product. Among the factors that can be easily modified, the type of bottle closure used during *tirage* may alter the physicochemical environment within the bottle, potentially affecting yeast metabolism, fermentation kinetics, and the subsequent chemical changes in the wine [12,13,14,15]. Crown caps are currently the most widely used closure system during secondary fermentation due to their ability to withstand high internal pressures, compatibility with automated bottling and disgorgement processes, and relatively low cost. Nevertheless, some producers continue to use *tirage*-cork stoppers, particularly in premium sparkling wine production, based on the belief that they may positively influence wine aging and sensory characteristics during bottle aging [16]. However, the extent to which different closures influence the biochemical and aroma profiles of sparkling wines during the secondary fermentation is still not fully understood.

Previous studies investigating bottle closure systems in traditional method sparkling wines have mainly focused on their influence during prolonged bottle aging, evaluating aspects such as phenolic composition, volatile profiles and sensory characteristics of the final wines [15,17]. By contrast, considerably less attention has been devoted to the potential influence of bottle closures during the active phase of secondary fermentation, when yeast metabolism is highly dynamic and major biochemical transformations occur.

Within this context, the present study aimed to monitor the compositional changes in a Portuguese sparkling wine during secondary fermentation, with a particular focus on free amino acids and volatile compounds. Furthermore, the effect of two bottle closure systems was evaluated throughout bottle fermentation. This approach provides new insights into the biochemical processes occurring during the *prise de mousse* and, unlike previous studies focused primarily on bottle aging, offers an integrated assessment of fermentation kinetics, amino acid metabolism and volatile compound development during the active phase of secondary fermentation.

## 2. Materials and Methods

### 2.1. Chemicals

The following volatile compounds were quantified: 1-hexanol (99.9%), 1-octanol (≥99%), 1-decanol (99.9%), 3-hexen-1-ol (98%), 3-methylbutan-1-ol (isoamyl alcohol, 98%), 2-phenylethan-1-ol (phenylethyl alcohol, 99%), 4-tert-butylcyclohexanol (98%), benzaldehyde (≥99.5%), phenylacetaldehyde (90%), decanal (95%), nonanal (≥95%), octanal (≥98%), ethyl butanoate (99%), ethyl hexanoate (99%), ethyl octanoate (99%), ethyl decanoate (≥98%), ethyl 2-methylbutanoate (99%), ethyl isobutanoate (≥98%), ethyl isovalerate (98%), ethyl heptanoate (≥98%), ethyl nonanoate (≥98%), diethyl succinate (≥99%), hexyl acetate (98%), 3-methylbutyl acetate (isoamyl acetate, ≥99%), 2-phenylethyl acetate (phenylethyl acetate, 99%), 2-heptanone (99%), 2-nonanone (97%), 2-undecanone (97%), 2,4-di-tert-butylphenol (99%), a-pinene (99%), b-linalool (80%), linalool oxide (97%), 1,8-cineole (99%), 1,4-cineole (98%), camphor (99%), limonene (99%), α-ionone (≥85%), b-ionone (≥85%), b-damascenone (≥98%), 1,1,6-trimethyl-1,2-dihydronaphthalene (99.5%), and a-terpineol (95%).

All chemicals used were purchased from Sigma-Aldrich (Madrid, Spain), except for ethanol (99.9%), which was obtained from ERBA Reagents (Val de Reuil, France).

### 2.2. Sparkling Wines and Closures

A Portuguese base wine from the Távora-Varosa region, produced in 2024, was filtered, stabilized, and bottled in November 2025. The wine consisted of a blend of two monovarietal wines from the Touriga Nacional and Touriga Franca grape varieties, in equal proportions (50% *v*/*v* each). The main oenological parameters of the base wine are reported in Table 1.

Prior to bottling, the tirage liqueur and a commercial *Saccharomyces cerevisiae* var. *bayanus* starter culture (DV10, Proefnol, Champagne, France; CIVC-approved) were added to the base wine in a homogenization tank. The mixture was continuously stirred to ensure a homogeneous distribution of yeast cells throughout the wine before bottling. During *tirage*, the wine was bottled using two different closure systems: (i) a standard 29 mm crown cap with high oxygen permeability (0.95 mg/year) [18]; and (ii) a dedicated *tirage*-cork stopper composed of an agglomerated cork body with two natural cork discs.

**Figure 1:**
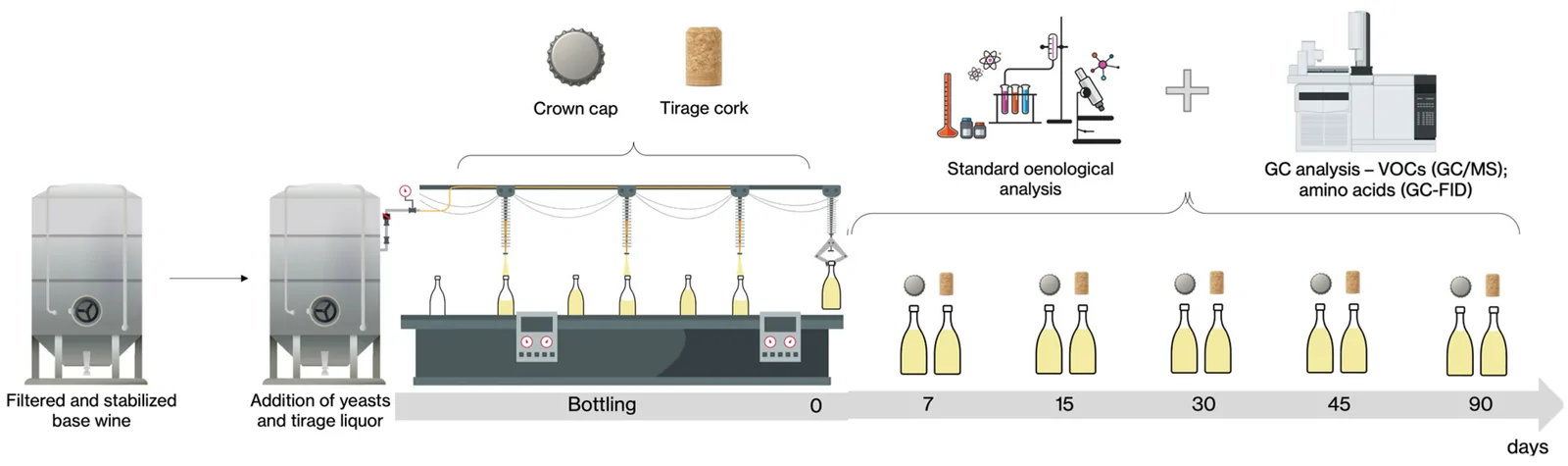
Experimental design and sampling timeline, illustrating the two closure systems used during *tirage* and the sampling points at 0, 7, 15, 30, 45, and 90 days.

All bottles were stored in the producer’s cellar under identical conditions. Samples were collected after 0, 7, 15, 30, 45, and 90 days in order to perform standard oenological analyses to monitor fermentation progress and to evaluate the progression of VOCs during the secondary fermentation. All analyses were performed directly on bottle samples without prior separation of yeast lees from the wine matrix. Prior to analysis, bottles were gently homogenized to ensure representative sampling. At each sampling point, one bottle from each closure system was provided by the producer and analyzed. Owing to the destructive nature of the analytical procedures, each bottle was analyzed only once, and bottle-to-bottle variability was therefore not evaluated in the present study. All analytical determinations were performed in triplicate, and the reported values correspond to the mean of three analytical replicates. The experimental design and sampling timeline are illustrated in Figure 1.

### 2.3. Standard Oenological Analysis

**Figure 2:**
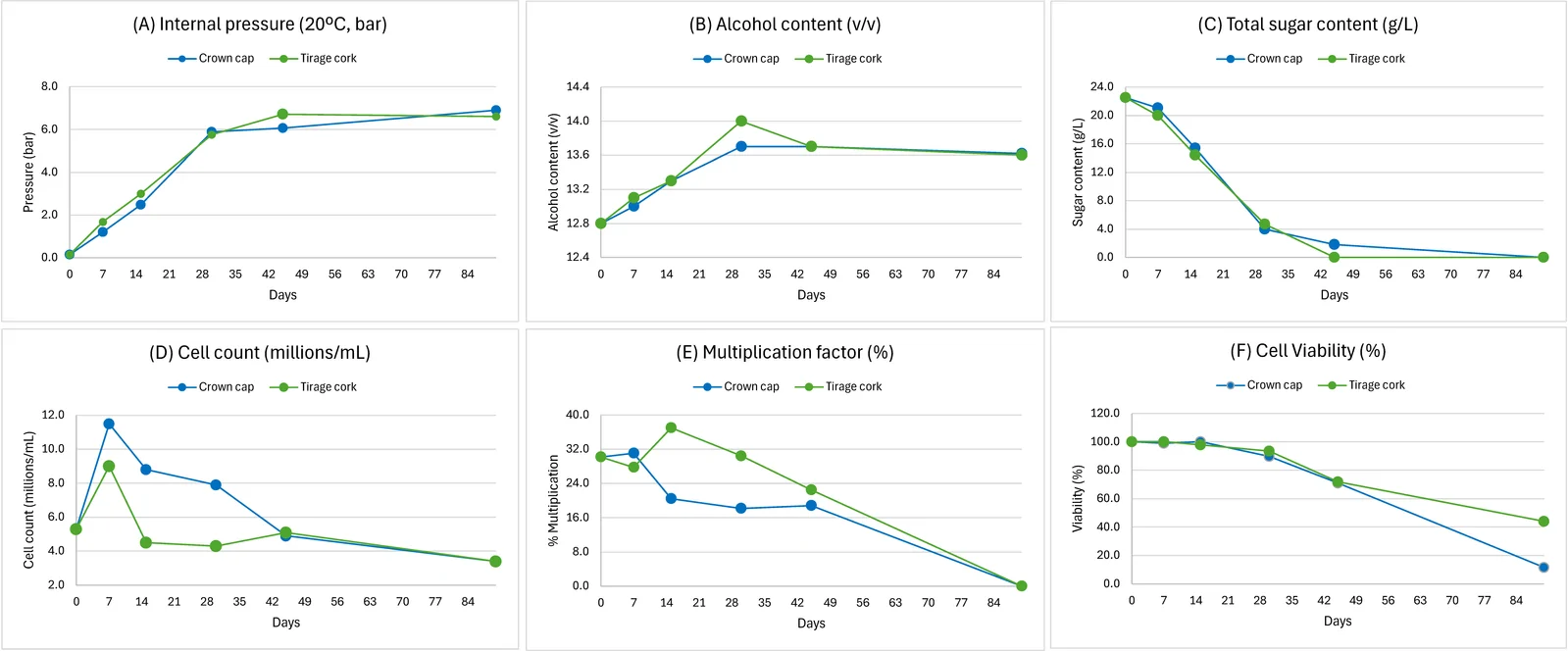
Temporal evolution of the main oenological parameters—(**A**) internal pressure (20 °C, bar); (**B**) alcohol content (*v*/*v*); (**C**) total sugar content (g/L); (**D**) cell count (in millions/mL); (**E**) multiplication factor (%); and (**F**) cell viability (%). Data are presented as the mean of three analytical replicates obtained from one bottle per closure system and sampling time.

All samples were analyzed in the cellar laboratory in order to monitor the evolution of the secondary fermentation. Internal pressure was measured using an aphrometer, and the recorded values were corrected to 20 °C. Total sugar content was determined using an enzymatic assay kit based on the Rebelein method (GAB System, Barcelona, Spain). Density was measured with a digital densimeter (DMA™ 35, Anton Paar, Graz, Austria). pH and total acidity were determined using a pH meter coupled to an automatic titration system (Titromatic 2S, Crison, Allela, Spain). Turbidity was assessed using a turbidimeter (Turbidirect, Lovibond, Dortmund, UK). Yeast populations were quantified by manual counting under a light microscope (Leitz Biomed, Leica Microsystems, Wetzlar, Germany) using a Neubauer improved counting chamber (DIN 12 847 standard; Paul Marienfeld GmbH & Co. KG, Lauda-Königshofen, Germany; chamber depth: 0.1 mm; grid area: 0.0025 mm^2^). Cell viability was determined by methylene blue staining. Prior to counting, samples were mixed with methylene blue solution and examined using a Neubauer improved counting chamber. Viable cells remained unstained, whereas non-viable cells stained blue. Cell viability was expressed as the percentage of viable cells relative to the total number of cells counted (viable plus non-viable cells). Additionally, samples were analyzed using an OenoFoss™ 2 (Foss, Hillerød, Denmark) for rapid multiparametric characterization and comparative analysis. For research purposes, mean values of the obtained results are presented in Figure 2 of the Results Section.

### 2.4. Analysis of Amino Acids by GC–FID

The amino acids present in sparkling wine samples were analyzed by gas chromatography using the Phenomenex EZ:Faast™ amino acid analysis kit (Phenomenex, Torrance, CA, USA), based on the method previously described elsewhere with slight modifications [19]. The kit includes two amino acid standard solutions, a solid-phase extraction column, the internal standard solution, and all reagents required for extraction and derivatization. Standard solution 1 consists of a mixture of 23 amino acids: α-aminoadipic acid (AAA), a-aminobutyric acid (ABA), alanine (ALA), allo-isoleucine (AILE), aspartic acid (ASP), b-aminoisobutyric acid (βAIB), cystine (C–C), glutamic acid (GLU), glycine (GLY), histidine (HIS), 4-hydroxyproline (HYP), isoleucine (ILE), leucine (LEU), lysine (LYS), methionine (MET), ornithine (ORN), phenylalanine (PHE), proline (PRO), sarcosine (SAR), serine (SER), threonine (THR), tyrosine (TYR), and valine (VAL). Standard solution 2 contains amino acids unstable under acidic conditions, namely asparagine (ASN), glutamine (GLN), and tryptophan (TRP).

The extraction procedure was identical for all samples and standards and followed previously described protocols [19,20]. Briefly, 200 μL of sonicated wine sample was mixed with 100 μL of the norvaline internal standard solution in a glass preparation vial. Amino acids were isolated by solid-phase extraction using the sorbent tip supplied with the kit, washed with 200 μL of 1-propanol/H_2_O washing solution, and dried by drawing air through the sorbent. Elution was performed using 200 μL of freshly prepared elution medium obtained by mixing an aqueous NaOH solution with a 1-propanol/3-picoline solution (3:2, *v*/*v*). The sorbent particles were then transferred to the reaction vial, and amino acids were derivatized by sequential addition of the propyl chloroformate-based derivatization reagent, the organic extraction solution, and dilute HCl, according to the manufacturer’s instructions. After phase separation, the upper organic layer containing the derivatized amino acids was collected for GC analysis.

Calibration curves were established for all amino acids, yielding correlation coefficients (R^2^) higher than 0.992. Analyses were performed using an Agilent 6890 gas chromatograph equipped with a flame ionization detector (FID) (Agilent Technologies, Santa Clara, CA, USA) and a Zebron ZB-AAA column (10 m × 0.25 mm i.d.; Phenomenex, Torrance, CA, USA). The oven temperature was programmed from 110 °C to 320 °C at 30 °C min^−1^, resulting in a total run time of 7 min. The injector and detector temperatures were set at 250 °C and 300 °C, respectively. A 2.0 μL aliquot was injected in split mode (1:15), using hydrogen as the carrier gas at a constant flow rate of 1.1 mL min^−1^. All samples were analyzed in triplicate. Representative chromatograms of amino acid standards and sparkling wine samples are provided in Figure S1.

### 2.5. Analysis of Volatile Composition by HS/SPME-GC/MS

The volatile composition of sparkling wines was analyzed over time using an HS/SPME-GC/MS method adapted from Barros, P. et al. [21]. Each sample (10 mL) was placed in a 20 mL glass vial, spiked with 100 µL of 4-methyl-2-pentanol (internal standard), and incubated for 5 min at 45 °C using a CombiPAL autosampler (CTC Analytics, Zwingen, Switzerland). Extraction was performed using a 50/30 μm divinylbenzene/carboxen/polydimethylsiloxane (DVB/CAR/PDMS) fiber (Supelco Inc., Bellefonte, PA, USA) for 30 min at 45 °C with a shaking speed of 250 rpm. The fiber was thermally desorbed for 6 min at 250 °C.

Chromatographic separation was carried out using an EVOQ GC-TQ system (Bruker Daltonics, Bremen, Germany) equipped with a fused silica capillary column (SLB-5ms, 30 m × 0.25 mm i.d. × 0.25 μm film thickness; Supelco Inc., Bellefonte, PA, USA). High-purity helium (6.0 grade, Gasin, Leça da Palmeira, Portugal) was used as carrier gas at 1.0 mL/min, with all runs performed in splitless mode for 3 min. The oven program was: 40 °C for 2 min, ramped at 2.5 °C/min to 170 °C (held for 5 min), then increased at 50 °C/min to 300 °C. Mass spectrometry was performed in electron ionization (EI) mode (70 eV), with transfer line, ion source, and manifold temperatures set at 280, 250, and 41 °C, respectively. Full scan acquisition ranged from *m*/*z* 40 to 400 with a scan time of 100 ms. Samples were analyzed by semi-quantification using the internal standard. Analyses were performed in triplicate. Representative chromatograms of sparkling wine samples are provided in Figure S2.

### 2.6. Statistical Analysis

Statistical analyses were performed using XLSTAT software 2020.2.3 (Addinsoft, Paris, France). The effects of closure system and fermentation time, as well as their interaction, on amino acid and volatile compound concentrations were evaluated using two-way analysis of variance (two-way ANOVA). The results obtained are expressed in Table S1 for amino acids and Table S2 for volatile composition. Pearson’s correlation analysis was performed to evaluate the relationship between the depletion of amino acids involved in the Ehrlich pathway and the accumulation of their corresponding volatile compounds. The results are presented in Table S3. Correlation coefficients (r) and associated *p*-values were calculated. Since one bottle per closure system was analyzed at each sampling point, the experimental design did not include independent biological replicates. Therefore, analytical triplicates were used exclusively to assess analytical repeatability rather than biological variability. Consequently, the two-way ANOVA and Pearson correlation analyses should be interpreted as exploratory statistical tools intended to identify temporal trends and potential closure-associated patterns within the monitored fermentation, rather than as confirmatory statistical analyses.

## 3. Results

To evaluate the influence of different closure systems during *tirage*, the progression of the main physicochemical and chemical parameters was monitored throughout the active phase of the secondary fermentation.

### 3.1. Evolution of Oenological Parameters

The temporal changes of the main oenological parameters are illustrated in Figure 2, in which each graph summarizes the kinetic trends observed throughout the experimental period. The combined assessment of pressure, ethanol production, residual sugar consumption, and yeast dynamics provides a comprehensive overview of fermentation progress and wine behavior under the two closure systems evaluated.

The variation in internal pressure, alcohol content, and residual sugar concentration (Figure 2A–C) reflected the expected progression of secondary fermentation. As *tirage* sugars were consumed, alcohol content increased while residual sugar concentration progressively decreased. Simultaneously, carbon dioxide accumulation led to a gradual increase in bottle pressure, with the most pronounced changes occurring during the first month after bottling. Although both closure systems reached similar final alcohol concentrations and residual sugar levels, differences in fermentation kinetics were observed during the intermediate stages of fermentation. Bottles sealed with the *tirage*-cork exhibited a faster increase in pressure and alcohol content, reaching maximum values earlier than crown-capped bottles. However, these differences progressively diminished, resulting in comparable final compositions for both treatments.

Yeast population dynamics (Figure 2D–F) showed rapid cell proliferation during the first week, followed by a gradual decline as fermentation progressed. Crown cap-fermented wine reached the highest total cell concentration during the active phase of fermentation, exhibiting a pronounced population peak at day 7. In contrast, *tirage*-cork-fermented wine displayed higher multiplication throughout most of the monitored period. Cell viability, defined as the proportion of membrane-intact cells determined by methylene blue staining, progressively decreased under both closure systems as fermentation advanced. Although crown-capped wine maintained higher viability during the initial stages, these differences became less evident over time, and both treatments converged toward similar viability values by the end of the monitored period.

At day 90, both closure systems reached comparable residual sugar concentrations, alcohol levels, pressure values, and yeast viability, indicating the completion of secondary fermentation under both sealing conditions.

### 3.2. Evolution of Free Amino Acids

Changes in free amino acid concentrations during the 90-day bottle fermentation were explored using a two-way ANOVA considering closure type, fermentation time, and their interaction as fixed factors. Because amino acids exhibited markedly different initial concentrations, results were expressed as the variation relative to the average concentration at day zero. This normalization minimized baseline differences among compounds and allowed direct comparison of their temporal progression throughout bottle fermentation. Exploratory statistical analysis suggested that fermentation time was the main factor associated with amino acid variability, whereas closure-related effects were generally more limited and compound-dependent. Several amino acids also displayed distinct temporal trajectories depending on the closure system employed, suggesting closure-associated differences in the timing and magnitude of the observed changes.

#### 3.2.1. Effect of Fermentation Time and Closure Type on Amino Acid Concentrations

**Figure 3:**
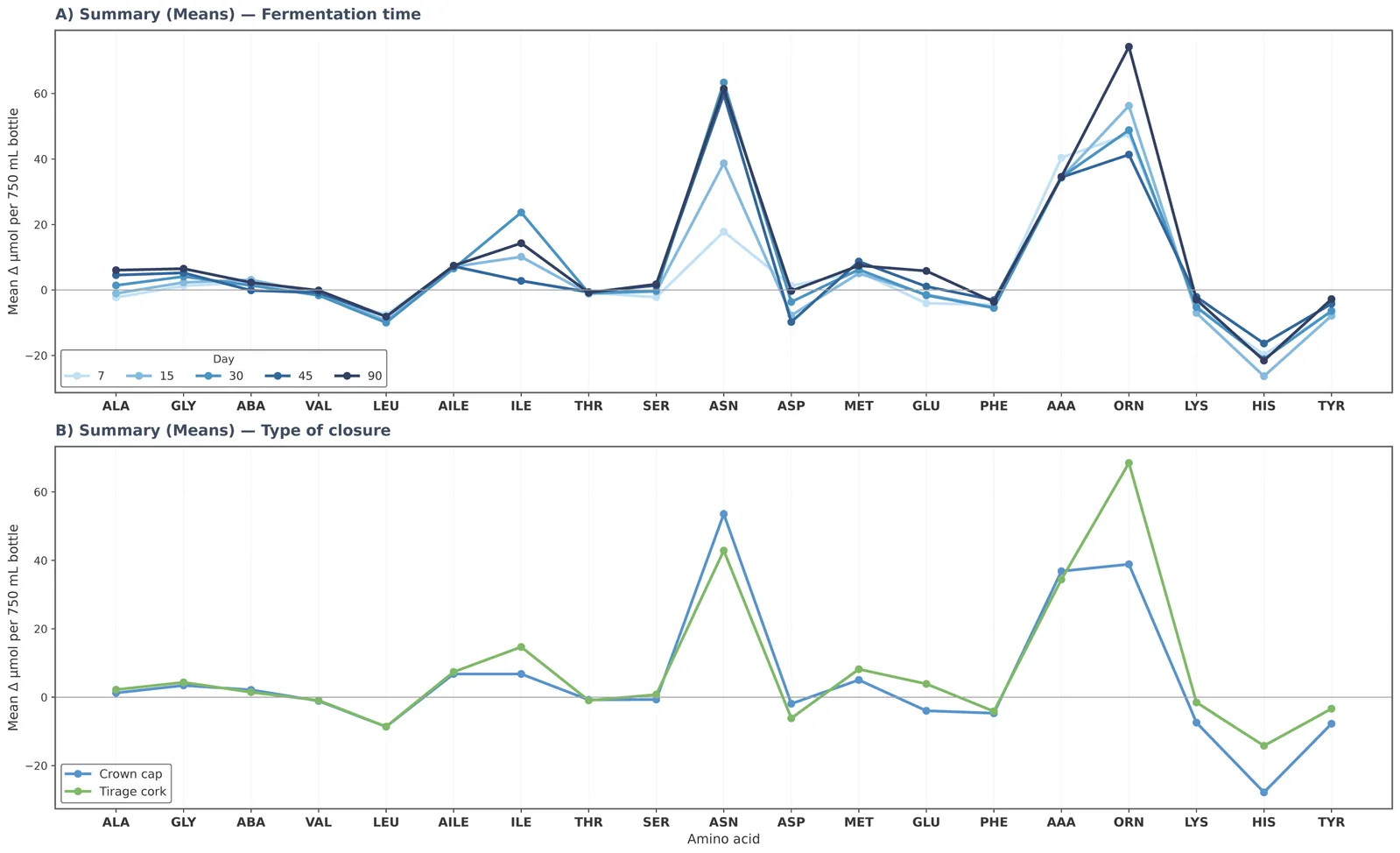
Mean variation (Δ µmol/bottle) of the amino acids quantified, summarized by time (**A**) and type of closure (**B**). In (**A**), shades of blue indicate the sampling time, from day seven (lightest blue) to day 90 (darkest blue). In (**B**), blue lines represent crown cap samples and green lines represent *tirage*-cork samples. Data are presented as the mean of three analytical replicates obtained from one bottle per closure system and sampling time.

Figure 3A illustrates the main effect of fermentation time on free amino acid concentrations, highlighting substantial temporal changes for most of the quantified amino acids during bottle fermentation. Relative to day zero, ASN, AAA, GLU, and ORN exhibited the largest positive variations among the amino acids monitored, although each compound followed a distinct temporal profile. ASN reached its highest variation during the intermediate stages of bottle fermentation, whereas GLU and ORN showed a more progressive increase over time. In contrast, ASP and HIS displayed the greatest negative variations relative to the initial composition, indicating net consumption throughout fermentation. Overall, the largest compositional changes were observed between 30 and 45 days, after which the amino acid profile tended to stabilise, although several compounds still exhibited measurable variations at day 90.

The effect of closure type on amino acid evolution is shown in Figure 3B. Although the overall pattern of variation was similar between closure systems, differences in the magnitude of the responses were observed for several amino acids. *Tirage*-cork generally exhibited greater positive variations for compounds such as ORN, ILE, and GLU, whereas crown-capped bottles showed higher ASN accumulation together with more pronounced negative variations for HIS and LYS. Despite these differences, fermentation time appeared to be the main factor associated with amino acid changes, whereas closure-associated differences were mainly observed in the magnitude rather than in the direction of the responses.

#### 3.2.2. Interaction Between Fermentation Time and Closure Type on Amino Acid Concentrations

**Figure 4:**
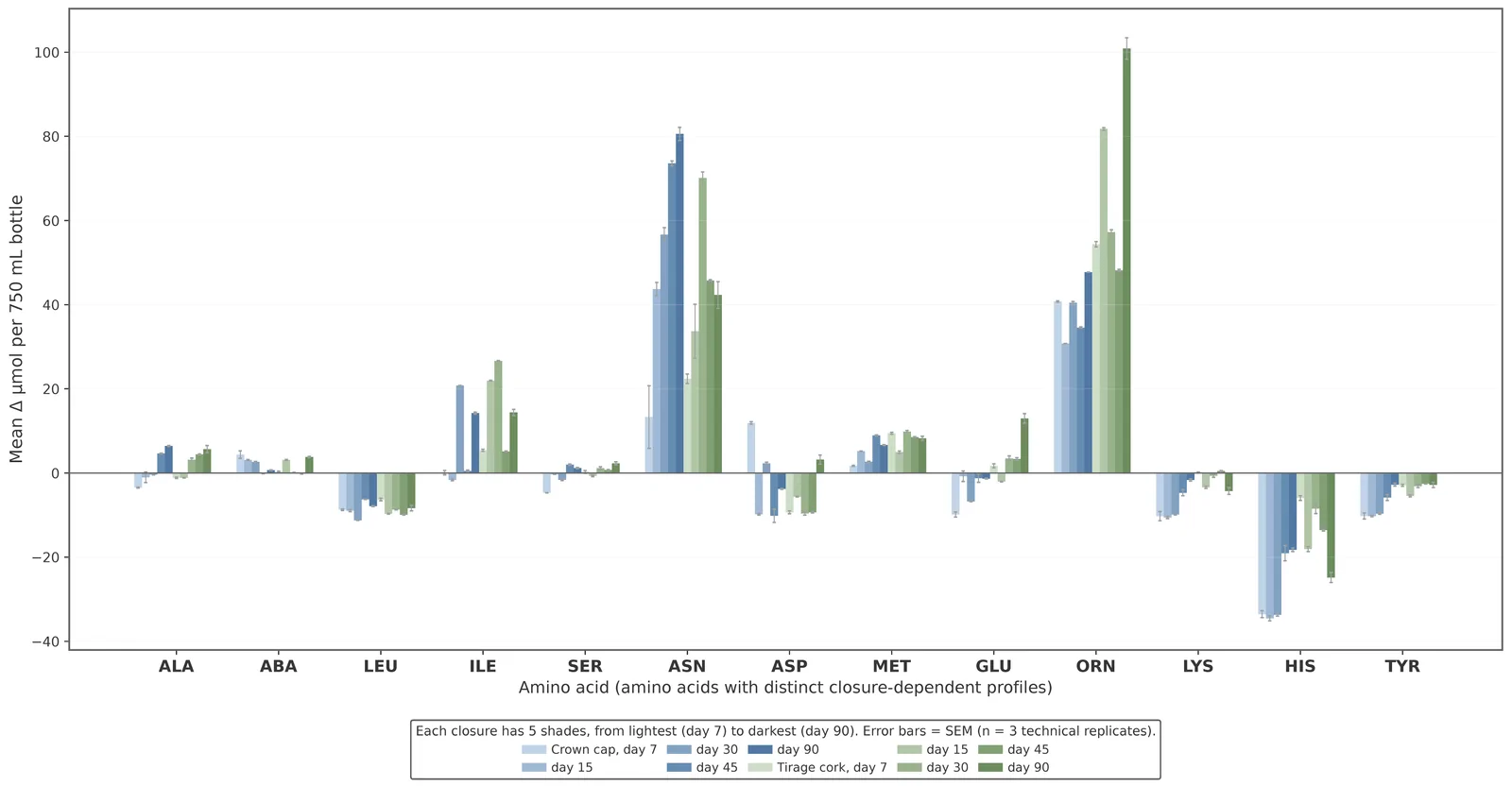
Mean variation (Δ µmol/bottle) of amino acids exhibiting distinct closure-dependent temporal evolution patterns (closure type × fermentation time interaction). Each closure has five shades, from day seven (lightest) to day 90 (darkest). Crown caps are represented in blue, and *tirage*-cork is represented in green. Data are presented as the mean of three analytical replicates obtained from one bottle per closure system and sampling time.

As expected, the interaction between closure type and fermentation time revealed that the temporal changes of several amino acids differed according to the sealing system employed (Figure 4). While the overall trajectories remained comparable among treatments, differences in the magnitude and timing of the observed variations were detected between closure systems. The greatest divergence among closure systems was observed during the intermediate stages of bottle fermentation, particularly between 30 and 45 days, when several amino acids exhibited distinct accumulation or consumption patterns between closure systems.

### 3.3. Evolution of Volatile Organic Compounds

A total of 39 volatile organic compounds were semi-quantified by HS/SPME-GC/MS analysis in order to understand how these compounds behave during the most active phase of fermentation. Of these, 29 compounds were quantified above the limit of quantification and included in the statistical analysis, while the remaining compounds were consistently below the quantification limit and were therefore excluded from further evaluation.

Similar to the amino acid analysis, changes in volatile compound concentrations during the 90-day bottle fermentation were explored using a two-way ANOVA considering closure type, fermentation time, and their interaction as fixed factors. Because volatile compounds exhibited markedly different initial concentrations and concentration ranges, results were expressed as the variation relative to the average concentration at day zero (Δ). This normalization minimized baseline differences among compounds and facilitated comparison of their temporal progression throughout bottle fermentation. Exploratory statistical analysis suggested that fermentation time was the main factor associated with volatile compound variability, whereas closure-related effects were generally more limited and compound-dependent.

#### 3.3.1. Effect of Fermentation Time and Closure Type on Volatile Composition

**Figure 5:**
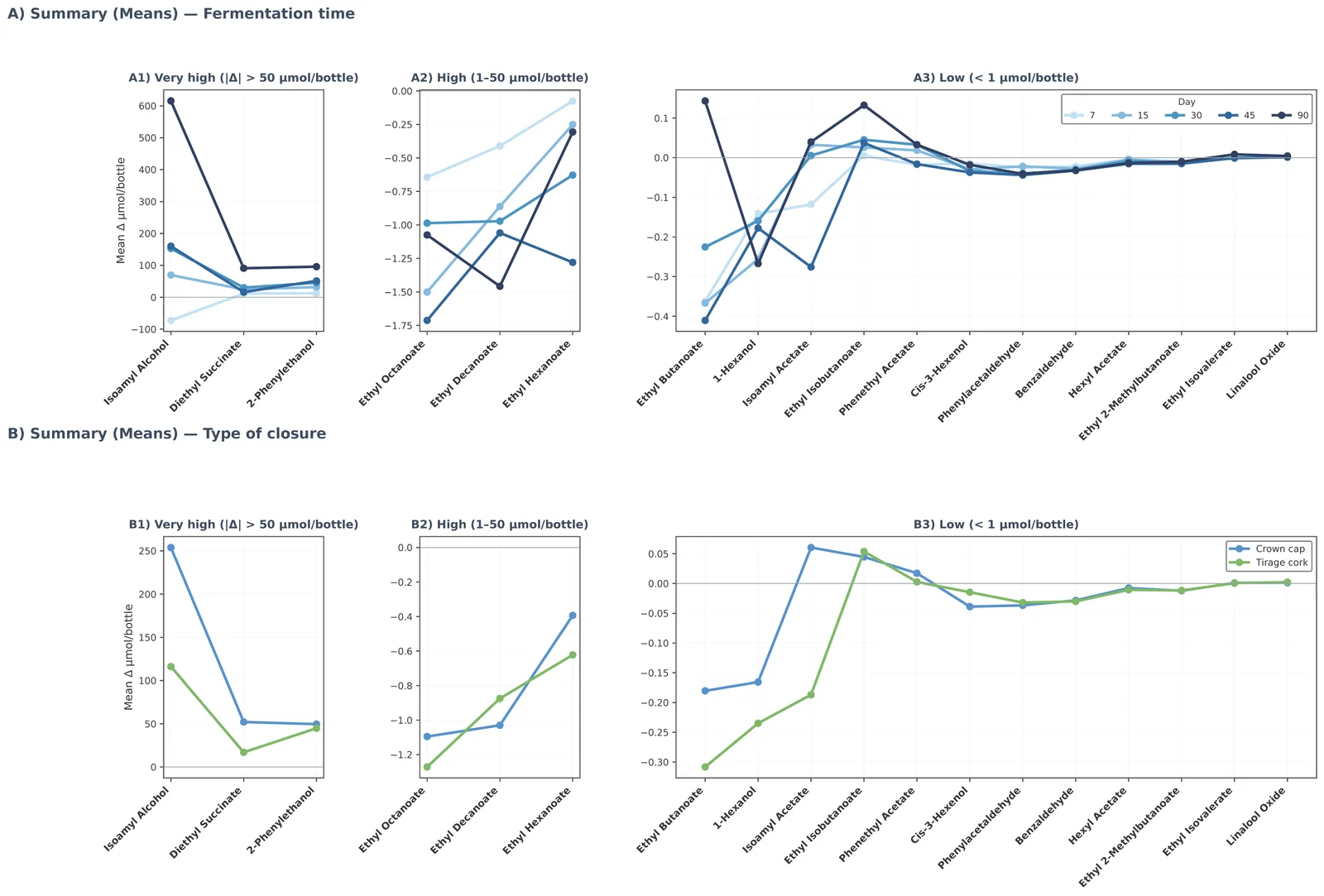
Mean variation (Δ µmol/bottle) of volatile compounds grouped according to the magnitude of their variation relative to day zero. (**A**) Main effect of fermentation time; (**B**) Main effect of closure type. Volatile compounds were grouped according to the magnitude of their variation relative to day zero as very high (|Δ| > 50 µmol/bottle), high (1–50 µmol/bottle), and low (|Δ| < 1 µmol/bottle). In (**A**), shades of blue indicate the sampling time, from day seven (lightest blue) to day 90 (darkest blue). In (**B**), blue lines represent crown cap samples and green lines represent *tirage*-cork samples. Data are presented as the mean of three analytical replicates obtained from one bottle per closure system and sampling time.

To facilitate visualization and interpretation, volatile compounds were grouped according to the magnitude of their variation relative to day zero and classified as very high (|Δ| > 50 µmol/bottle), high (1–50 µmol/bottle), and low (|Δ| < 1 µmol/bottle) abundance compounds. Thresholds (|Δ| > 50, 1–50 and |Δ| < 1 µmol/bottle) were defined solely to improve graphical readability by separating compounds with markedly different ranges of variation. Their temporal changes and the effect of closure type are presented separately in Figure 5.

The main effect of fermentation time on volatile behavior is presented in Figure 5A. The most pronounced temporal changes were observed for fermentation-derived compounds, particularly higher alcohols and esters. Isoamyl alcohol and 2-phenylethanol displayed progressive increases throughout bottle fermentation, whereas several esters exhibited negative variations relative to their initial concentrations. Among compounds showing intermediate variations, phenylacetaldehyde, benzaldehyde, and 2-nonanone generally increased during fermentation, while terpene-derived compounds exhibited comparatively smaller changes.

The largest compositional changes occurred between 30 and 45 days, coinciding with the period during which the most pronounced modifications in amino acid composition were also observed.

The effect of closure type on volatile development is presented in Figure 5B. Although closure-associated differences were generally less pronounced than the temporal variations observed throughout fermentation, noticeable differences in magnitude were evident for several compounds exhibiting the largest overall changes. In particular, isoamyl alcohol, ethyl acetate, ethyl lactate, diethyl succinate, phenylacetaldehyde, benzaldehyde, 1-hexanol and 2-nonanone displayed distinct average variations between closure systems, whereas 2-phenylethanol showed comparatively similar responses under both closures. Among the compounds exhibiting lower overall variation, cis-3-hexenol was the only compound showing a clear closure-dependent response. Overall, closure-associated differences were primarily observed in the magnitude of volatile compound variation rather than in the direction of the observed changes.

#### 3.3.2. Interaction Between Fermentation Time and Closure Type on Volatile Composition

**Figure 6:**
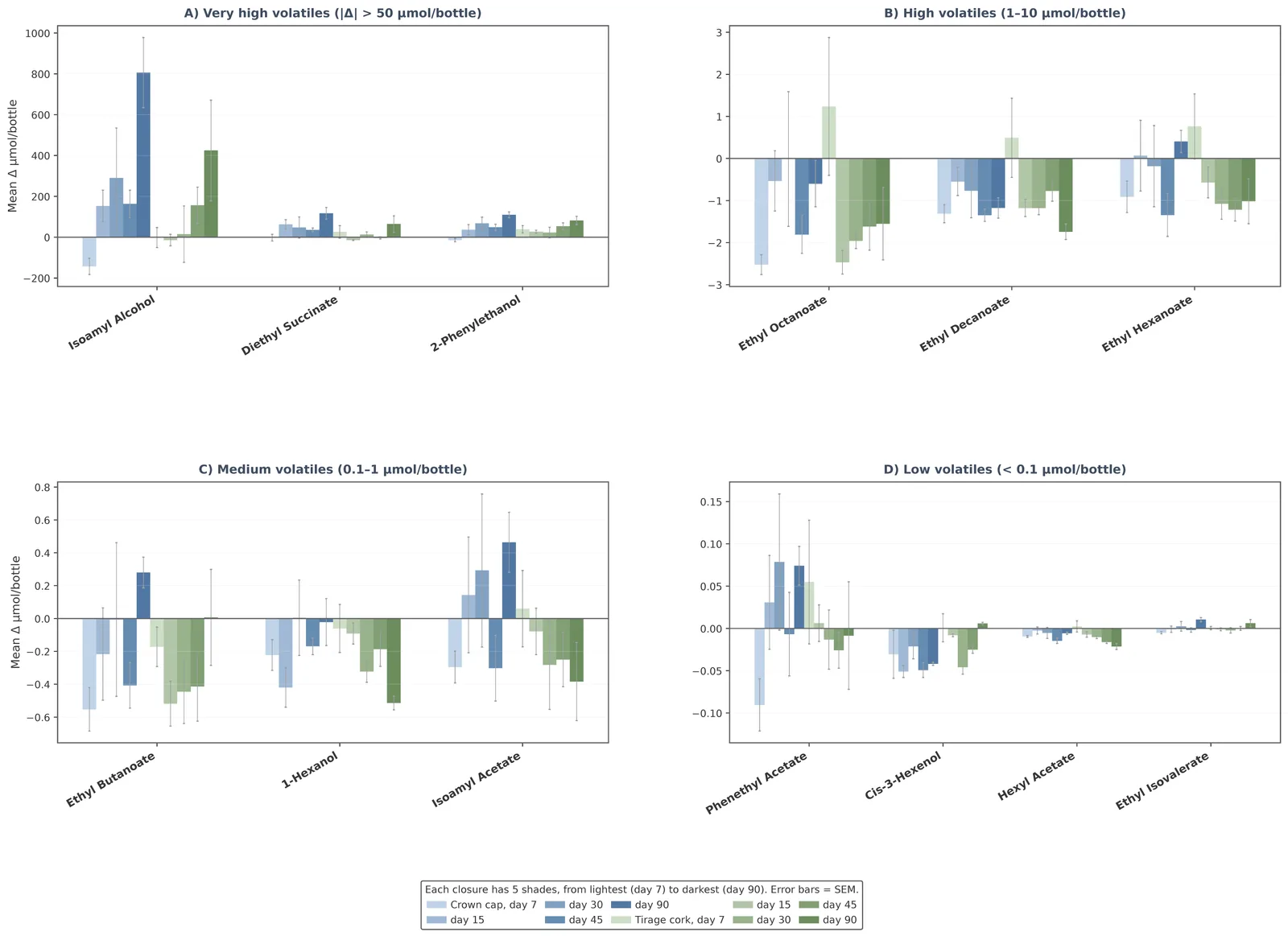
Mean variation (Δ µmol/bottle) of volatile compounds exhibiting distinct closure-dependent temporal evolution patterns. Volatile compounds are grouped according to the magnitude of their variation relative to day zero: (**A**) very high, (**B**) high, (**C**) medium, and (**D**) low-abundance compounds. Blue and green colors represent crown cap and tirage-cork closures, respectively, while progressive color shades indicate sampling time from day 7 (lightest) to day 90 (darkest). Data are presented as the mean of three analytical replicates obtained from one bottle per closure system and sampling time.

The interaction between closure type and fermentation time for volatile compounds is presented in Figure 6. Thresholds were defined solely to improve graphical readability by separating compounds with markedly different ranges of variation.

Several compounds exhibited distinct closure-dependent temporal evolution patterns, indicating that the effect of closure varied throughout the course of secondary fermentation rather than remaining constant over time.

The most pronounced differences were observed among compounds exhibiting the largest concentration changes during fermentation, particularly higher alcohols and fermentation-derived esters. For these compounds, divergence between closure systems became most evident during the intermediate stages of bottle fermentation, corresponding to the period of greatest metabolic activity previously identified from both the oenological parameters and amino acid dynamics.

Compounds exhibiting intermediate and low abundance generally showed more moderate closure-dependent responses, although temporal trajectories occasionally differed between closure systems. In most cases, differences between wines with crown cap and *tirage*-cork were less evident at the beginning and end of the experimental period, whereas greater divergence was observed between 30 and 45 days.

Overall, these interaction patterns suggest closure-associated differences in the temporal evolution of a limited number of volatile compounds, with responses that were compound-specific and time-dependent.

### 3.4. Relationship Between Amino Acid Consumption and Volatile Compound Production

**Figure 7:**
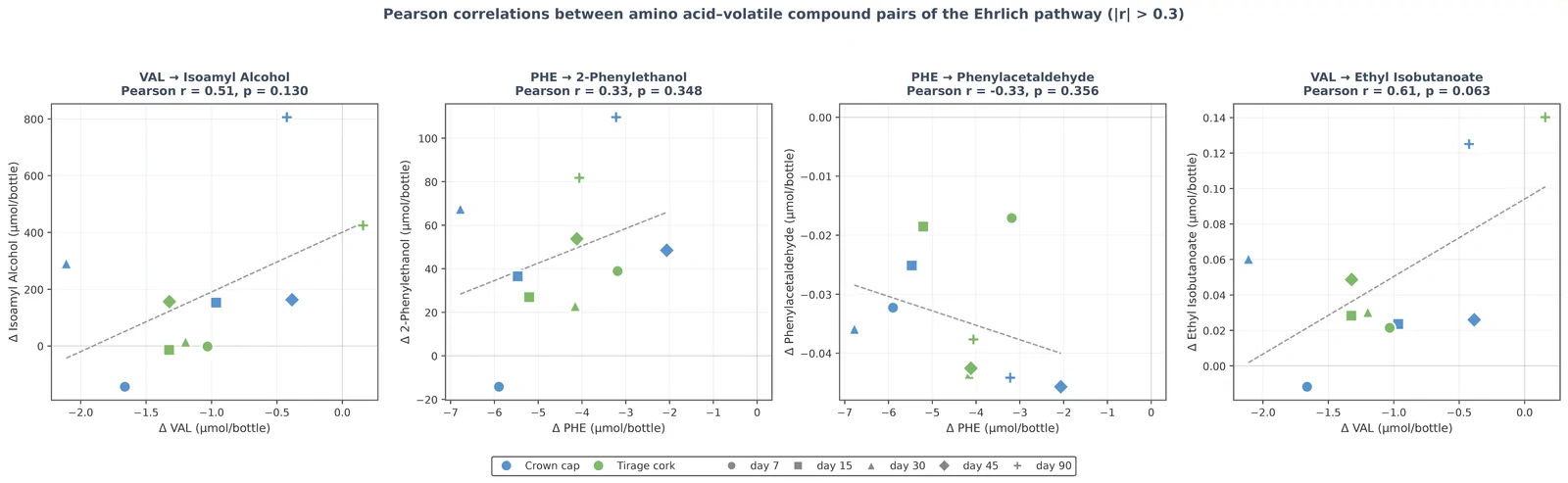
Scatter plots show the relationships between changes relative to day zero (Δ) for VAL and isoamyl alcohol, PHE and 2-phenylethanol, PHE and phenylacetaldehyde, and VAL and ethyl isobutanoate. Dashed lines represent the fitted linear regressions. Blue and green colors represent crown cap and tirage-cork closures, respectively, while symbols indicate sampling time (○ day 7; □ day 15; △ day 30; ◇ day 45; + day 90). Each point corresponds to the mean of three analytical replicates obtained from one bottle per closure system and sampling time.

To further explore the relationship between nitrogen metabolism and aroma formation during secondary fermentation, Pearson correlation analyses were performed between selected amino acids and their corresponding volatile compounds associated with the Ehrlich pathway (Figure 7 for correlations |r| > 0.3 and Figure S3 for correlations |r| < 0.3).

Both positive and negative associations were observed among the amino acid-volatile compound pairs evaluated. The strongest positive correlations were detected between VAL and ethyl isobutanoate (r = 0.61, *p* = 0.063) and between VAL and isoamyl alcohol (r = 0.51, *p* = 0.130). Weaker relationships were observed between PHE and 2-phenylethanol (r = 0.33, *p* = 0.348) and between PHE and phenylacetaldehyde (r = −0.33, *p* = 0.356).

Although none of the correlations reached statistical significance, several moderate associations were observed between amino acids and fermentation-derived volatile compounds. These relationships should be interpreted as exploratory observations and do not demonstrate direct precursor–product relationships. The distribution of the data points illustrates the variability of these associations across closure systems and sampling times but should not be interpreted as evidence of causal relationships.

## 4. Discussion

The present study provides a time-resolved characterization of the progression of basic oenological parameters, free amino acids, and volatile compounds during the first 90 days of the secondary fermentation of traditional method sparkling wines produced under two different closure systems. To the best of our knowledge, this is the first study to comprehensively investigate the metabolic dynamics of this active fermentation stage under different bottle closure systems.

The evolution of the basic oenological parameters followed the characteristic pattern described for traditional method sparkling wines, with progressive sugar consumption accompanied by ethanol production and CO_2_ accumulation [22,23]. However, despite similar final compositions, cork-fermented wines displayed faster sugar consumption and pressure development during the active phase of fermentation. These differences were particularly interesting when considered alongside yeast population dynamics. While crown cap-fermented wines reached higher maximum yeast populations, cork-fermented wines exhibited higher multiplication factors and more rapid fermentative progression. Such observations suggest that fermentation performance was also influenced by differences in yeast physiological behavior. Previous studies have shown that relatively small modifications in the physicochemical environment surrounding yeast cells may influence growth patterns, metabolic activity and subsequent wine profile [17,24,25,26]. Likewise, Jolly, N. et al. and Minnaar, P. et al. reported compositional and sensory differences between sparkling wines produced under distinct closure systems, supporting the view that bottle closure may influence fermentation behavior from the earliest stages of the secondary fermentation rather than only during subsequent aging [12,14,27].

These observations are particularly relevant when considered alongside the progression of free amino acids, which revealed substantial modifications in central nitrogen metabolism during the same period. Fermentation time emerged as the dominant factor governing amino acid changes, indicating that nitrogen metabolism remained highly dynamic throughout bottle fermentation. The largest changes were observed for ASN, ORN, HIS, AAA, GLU and ASP, suggesting that secondary fermentation involved extensive reorganization of intracellular nitrogen fluxes rather than simple amino acid consumption. In addition, several branched-chain and aromatic amino acids, including VAL, LEU, ILE and PHE, also exhibited temporal variations throughout fermentation. Although less pronounced than those observed for the central nitrogen metabolites, these changes are particularly relevant because these amino acids act as direct precursors of aroma-active compounds through the Ehrlich pathway and therefore provide a mechanistic link between nitrogen metabolism and volatile compound formation [11].

ASN is widely recognized as one of the principal nitrogen storage compounds in yeast cells, allowing excess nitrogen to be temporarily conserved and subsequently redistributed according to cellular requirements [28,29]. Liu, D. et al. identified glutamic acid and aspartic acid as central intermediates in nitrogen redistribution pathways and highlighted their importance in maintaining nitrogen homeostasis under changing nutritional conditions [30]. In the present study, ASP decreased progressively while ASN and GLU increased throughout fermentation. Although these data alone cannot reveal the precise metabolic routes involved, the coordinated evolution of these amino acids suggests that active nitrogen redistribution mechanisms remained operative during the fermentation period.

Another amino acid displaying a remarkable accumulation pattern was ORN. Unlike ASN, whose dynamics primarily reflect nitrogen storage, ORN is more closely associated with the regulation and recycling of intracellular nitrogen [31,32]. Messenguy, F. et al. described ORN as an intermediate connecting glutamic acid metabolism, arginine biosynthesis and urea-cycle-related processes in *Saccharomyces cerevisiae* [33]. Although arginine and urea were not quantified in the present study, the accumulation of ORN indicates that secondary fermentation was accompanied by substantial adjustments in cellular nitrogen management. This interpretation aligns with Zhao, X. et al., who highlighted the close relationship between nitrogen metabolism and urea-associated processes in yeast. Together with the changes observed for ASN, ASP and GLU, the behavior of ORN reinforces the view that secondary fermentation involves active reorganization of intracellular nitrogen resources rather than simple amino acid consumption [34].

The increase observed for AAA provides additional support for the existence of broader metabolic adjustments involving nitrogen metabolism. Although AAA has received relatively little attention in sparkling wine studies, it is recognized as an intermediate of lysine biosynthesis, a pathway that competes for nitrogen resources and is closely interconnected with central nitrogen metabolism. Marsit, S. et al. reported that increased glutamate availability may be associated with the accumulation of both AAA and ORN, reflecting coordinated regulation of interconnected nitrogen-dependent processes [35].

In contrast, HIS exhibited a progressive decrease throughout fermentation. This amino acid plays important roles in protein synthesis, cellular growth and nitrogen metabolism and is frequently consumed during periods of active cellular activity. Bianchi, F. et al. reported substantial histidine utilization during yeast fermentation and associated this behavior with amino acid assimilation and metabolic adaptation [36].

Interestingly, closure-dependent differences were particularly evident for ASN, ORN and HIS. Crown cap-fermented wines exhibited greater ASN accumulation and more pronounced HIS depletion, whereas cork-fermented wines displayed markedly higher ORN concentrations at the same sampling points. The observed amino acid changes likely reflect the combined effects of yeast uptake, metabolic interconversion and intracellular nitrogen redistribution during the active phase of secondary fermentation. Autolytic release was considered unlikely to be a major contributor during most of the monitored period, given that the study focused on the active phase of bottle fermentation rather than prolonged lees aging. This pattern may be consistent with differences in nitrogen redistribution pathways involving the urea cycle and interconnected nitrogen metabolism in wines sealed with tirage cork. However, no direct metabolic or transcriptomic evidence was generated in the present study to confirm the underlying mechanisms, and this interpretation should therefore be considered as a possible explanation for the observed amino acid profiles. Although direct evidence regarding the role of closure type on amino acid evolution during sparkling wine secondary fermentation remains limited, the present results indicate that distinct amino acid profiles were observed between the two closure systems throughout fermentation.

The progression of volatile compounds revealed that these metabolic differences extended beyond amino acid metabolism and were accompanied by substantial modifications in aroma-related compounds. The most pronounced increases were observed for isoamyl alcohol, diethyl succinate and 2-phenylethanol, whereas several ethyl esters, including ethyl octanoate, ethyl decanoate and ethyl hexanoate, exhibited more dynamic behavior throughout fermentation. These compounds displayed an initial decrease relative to day zero followed by a gradual recovery during the intermediate stages of fermentation, although ethyl octanoate and ethyl decanoate subsequently declined again towards day 90. Such temporal patterns suggest that ester synthesis and degradation remained active throughout bottle fermentation, reflecting the continued metabolic activity of yeast cells under sparkling wine production conditions. However, the evolution of these compounds may also reflect closure-related phenomena, including differential aroma adsorption (scalping) and potential differences in oxygen transfer properties between the two closure systems, although no direct oxygen measurements were performed in the present study. Comparable trends have been reported during sparkling wine production and aging on lees, where continued yeast metabolism promotes the accumulation of certain higher alcohols and fermentation-derived compounds while modifying ester composition [37,38].

Among the compounds displaying the largest increases, 2-phenylethanol is particularly relevant because it is associated with floral and rose-like aroma attributes and is widely recognized as one of the principal products of phenylalanine catabolism through the Ehrlich pathway [39]. Likewise, phenylacetaldehyde contributes floral and honey-like notes and occupies an intermediate position in the conversion of PHE into 2-phenylethanol [40]. Interestingly, phenylacetaldehyde remained comparatively stable throughout fermentation despite the marked accumulation of 2-phenylethanol. This behavior is consistent with its role as a transient metabolic intermediate that is continuously produced and subsequently reduced to 2-phenylethanol rather than accumulating within the wine matrix. Isoamyl alcohol, derived primarily from branched-chain amino acid metabolism, contributes fermentative and fruity nuances and represents one of the most abundant higher alcohols in wine [11,41]. The accumulation of these compounds throughout secondary fermentation therefore suggests that the observed modifications in nitrogen metabolism were accompanied by changes in pathways directly associated with aroma formation.

Closure-associated differences were also evident for several volatile compounds, although these effects were generally smaller than the temporal changes associated with fermentation progress. The most noticeable differences were observed for isoamyl alcohol, diethyl succinate, ethyl butanoate, ethyl octanoate, ethyl hexanoate, 1-hexanol and particularly isoamyl acetate. Several of these compounds contribute fruity, fermentative and fresh aroma attributes that are important components of sparkling wine aroma. Interestingly, isoamyl acetate exhibited contrasting behavior under the two closure systems, showing net accumulation under crown cap conditions while progressively decreasing under *tirage*-cork [42]. Given the dynamic synthesis and hydrolysis of isoamyl acetate during fermentation, these differences are more likely to reflect transient metabolic responses rather than persistent differences in the aroma profile of the final sparkling wine.

Pearson correlation analysis provided an exploratory assessment of the relationships between amino acids and volatile compounds associated with the Ehrlich pathway. The strongest associations were observed between VAL and ethyl isobutanoate and between VAL and isoamyl alcohol, whereas weaker relationships were detected between PHE and 2-phenylethanol and between PHE and phenylacetaldehyde. However, the absence of strong precursor–product relationships agrees with several studies indicating that aroma development results from the integration of multiple metabolic processes rather than from the direct consumption of individual amino acid precursors alone. Scott, W. et al. reported that aroma production depends not only on precursor availability but also on yeast physiology, nitrogen regulation and fermentation conditions [43]. Similarly, Sun, N. et al. showed that relationships between amino acids and volatile compounds are frequently weak because multiple metabolic processes operate simultaneously and individual amino acids may contribute to several biochemical routes [7]. Tesařík, S. et al. likewise reported that changes in amino acid composition during sparkling wine secondary fermentation were not always directly reflected in the concentrations of the corresponding volatile compounds [22]. Collectively, these findings support the interpretation that amino acid availability represents only one of several factors governing aroma formation during bottle fermentation and that precursor concentration alone cannot fully explain the evolution of volatile compounds.

Most previous studies on traditional method sparkling wines have focused primarily on the effects of prolonged aging on lees, during which yeast autolysis dominates the compositional changes of the wine [5,25,44]. By contrast, the present work demonstrates that extensive modifications in oenological parameters, amino acid composition and volatile production already occur during the active phase of secondary fermentation, well before extended lees aging becomes the predominant process. Moreover, the distinct metabolic trajectories observed under crown cap and *tirage*-cork conditions suggest that the choice of closure system may be associated with differences in yeast metabolic behavior during bottle fermentation, ultimately contributing to distinct compositional trajectories. By integrating these complementary datasets within a common statistical framework, this study provides a comprehensive description of the biochemical events occurring during bottle fermentation and highlights the central role of yeast metabolism in shaping wine composition at this early stage.

Despite the robustness of the analytical methodology and the clear metabolic trends observed, some limitations should be acknowledged. The absence of direct measurements of dissolved oxygen and headspace gas composition prevents definitive attribution of the observed closure-associated differences to oxygen transfer properties. In addition, the limited biological replication reduces the strength of the statistical inference and therefore the reported statistical differences should be interpreted as exploratory rather than confirmatory. Consequently, although the observed trends provide valuable insight into the dynamics of amino acids and volatile compounds during secondary fermentation, confirmation through studies incorporating independent biological replication and direct oxygen measurements would further strengthen these findings.

Future studies integrating direct oxygen measurements, yeast viability, multiple commercial *Saccharomyces cerevisiae* strains, and extended monitoring throughout lees aging and post-disgorgement evolution would provide a more comprehensive understanding of the mechanisms underlying closure-associated metabolic responses and determine whether the differences observed during active secondary fermentation persist in the final sparkling wine. Overall, the present findings suggest that bottle closure should be considered an important oenological variable associated with differences in fermentation kinetics, nitrogen metabolism and aroma development during the active phase of secondary fermentation, opening new perspectives for optimizing sparkling wine production through targeted management of bottle fermentation conditions.

## 5. Conclusions

This study provides a comprehensive characterization of the active phase of secondary fermentation in traditional method sparkling wines by simultaneously evaluating fermentation kinetics, free amino acid metabolism and volatile compound progression under two bottle closure systems. The results showed that extensive biochemical modifications occur within the first 90 days after bottling, indicating that this stage contributes substantially to the establishment of the final chemical and aromatic profile of sparkling wines before prolonged lees aging becomes the dominant process.

Fermentation time was the principal factor governing metabolite dynamics, with the period between 30 and 45 days corresponding to the highest yeast metabolic activity. The contrasting evolution of ASN, ORN, AAA, GLU, ASP and HIS reflected active nitrogen metabolism throughout bottle fermentation, while the accumulation of higher alcohols and other fermentation-derived volatile compounds reinforced the close relationship between nitrogen metabolism and aroma development.

Closure-associated differences were also observed in fermentation kinetics and in the temporal changes of several amino acids and volatile compounds, suggesting distinct metabolic and compositional trajectories between the two closure systems during secondary fermentation. However, given the limited biological replication and the absence of direct oxygen measurements, these findings should be interpreted as exploratory and warrant confirmation through future studies incorporating independent biological replication and direct assessment of oxygen transfer.

The integration of oenological, metabolomic and aroma-related data represents one of the principal strengths of this work and highlights the importance of considering bottle closure as a technological factor potentially associated with the biochemical changes occurring during the active phase of secondary fermentation, rather than solely as a sealing system.

## Figures and Tables

**Table 1:** Oenological parameters of the base wine.

Oenological Parameters	Base Wine
Density (g/cm^3^)	0.9879
Alcohol content (*v*/*v*)	12.81
Total acidity (g/L)	4.41
Volatile acidity (g/L)	0.24
pH	3.26

## Data Availability

The original contributions presented in this study are included in the article/supplementary material. Further inquiries can be directed to the corresponding author.

## Supplementary Materials

The following supporting information can be downloaded at: https://www.mdpi.com/article/10.3390/microorganisms14081623/s1. Figure S1. Representative chromatograms of amino acid standards and sparkling wine samples obtained by GC–FID; Figure S2. Representative chromatograms of sparkling wine samples obtained by GC–MS; Table S1. Two-way ANOVA results for amino acid concentrations; Table S2. Two-way ANOVA results for volatile organic compounds (VOCs); Table S3. Pearson correlation coefficients between selected amino acid–volatile compound pairs involved in the Ehrlich pathway; Figure S3. Pearson correlations between amino acid–volatile compound pairs associated with the Ehrlich pathway exhibiting weak associations (|r| < 0.3). Scatter plots show the relationship between variation relative to day 0 (Δ) for the remaining precursor–product pairs not included in Figure 7. Symbols indicate closure type and sampling time.
