# Supplementary material for: Influence of Different Closure Systems on Yeast Metabolism During the Secondary Fermentation of Traditional Method Sparkling Wines
Source: Microorganisms. 2026 Jul 24;14(8):1623. doi: 10.3390/microorganisms14081623 (PMC13515494; doi:10.3390/microorganisms14081623)
Supplement: Supplementary file 1 [file microorganisms-14-01623-s001.zip › microorganisms-4439138-supplementary.pdf]

Supplementary information:

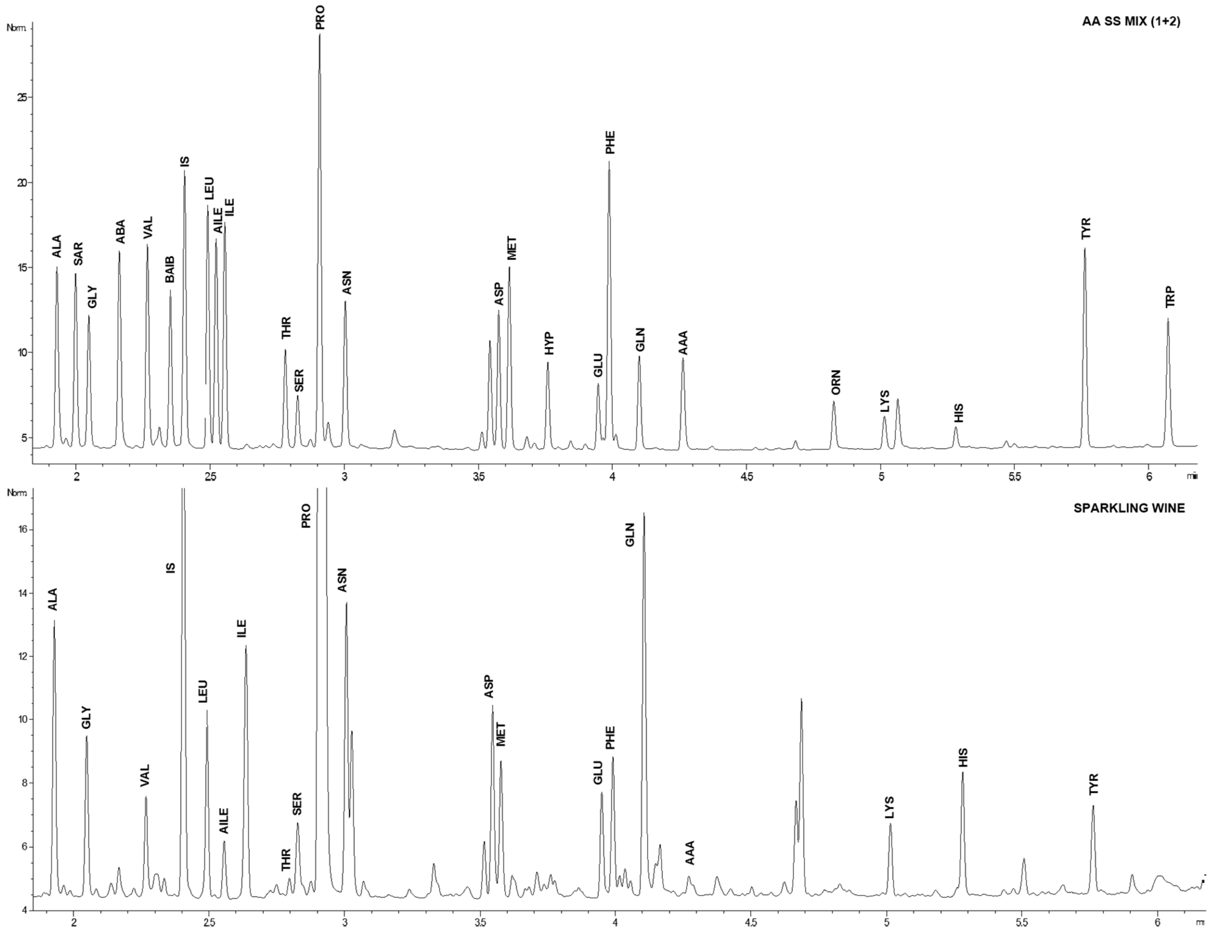

Figure S1. Representative chromatograms of amino acid standards and sparkling wine samples obtained by GC–FID.

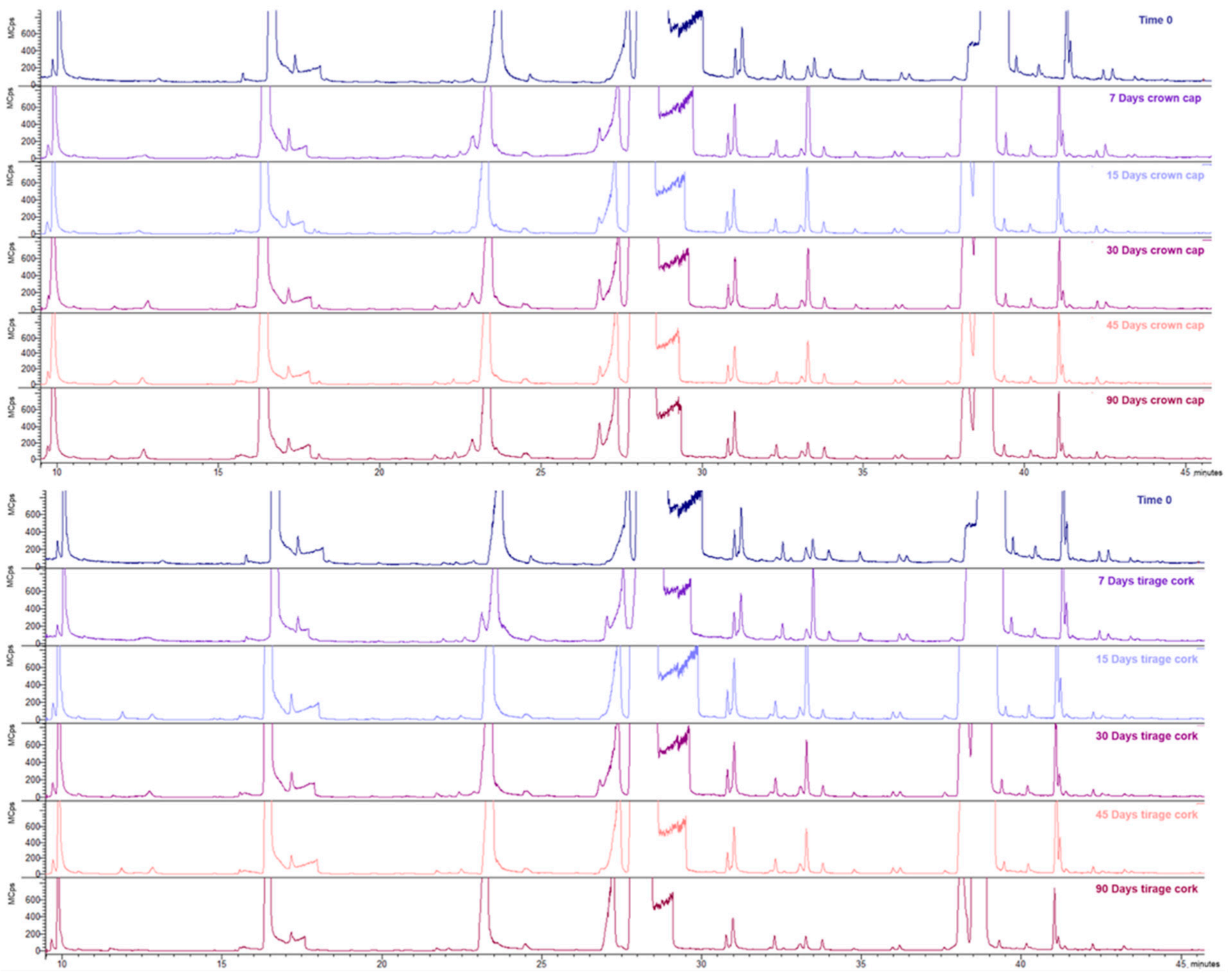

Figure S2. Representative chromatograms of sparkling wine samples obtained by GC–MS.

Table S1. Two-way ANOVA results for amino acid concentrations.

Amino acid	Type of closure		Days		Closure × Days		R^2
	p-value	sig	p-value	sig	p-value	sig	
ALA	0,0069	**	< 0.001	***	0,0012	**	0,9561
GLY	< 0.001	***	< 0.001	***	< 0.001	***	0,9652
ABA	0,0015	**	< 0.001	***	< 0.001	***	0,942
VAL	0,1171	ns	< 0.001	***	< 0.001	***	0,8856
LEU	0,7831	ns	< 0.001	***	< 0.001	***	0,9947
AILE	< 0.001	***	< 0.001	***	< 0.001	***	0,8859
ILE	< 0.001	***	< 0.001	***	< 0.001	***	0,9978
THR	0,1105	ns	0,1065	ns	0,0167	*	0,5763
SER	0,0438	*	< 0.001	***	< 0.001	***	0,9337
ASN	< 0.001	***	< 0.001	***	< 0.001	***	0,9493
ASP	< 0.001	***	< 0.001	***	< 0.001	***	0,979
MET	< 0.001	***	< 0.001	***	< 0.001	***	0,9869
GLU	< 0.001	***	< 0.001	***	< 0.001	***	0,9683
PHE	0,1031	ns	< 0.001	***	< 0.001	***	0,846
AAA	0,3391	ns	0,4643	ns	0,3285	ns	0,325
ORN	< 0.001	***	< 0.001	***	< 0.001	***	0,9965
LYS	< 0.001	***	< 0.001	***	< 0.001	***	0,9397
HIS	< 0.001	***	< 0.001	***	< 0.001	***	0,9655
TYR	< 0.001	***	< 0.001	***	< 0.001	***	0,9735

Table S2. Two-way ANOVA results for volatile organic compounds (VOCs).

VOCs	Type of closure		Days		Closure × Days		R^2
	p-value	sig	p-value	sig	p-value	sig	
Isoamyl Alcohol	0,1317	ns	0,0011	**	0,3683	ns	0,635
Cis-3-Hexenol	0,0055	**	0,3225	ns	0,0562	ns	0,5626
1-Octanol	0,3084	ns	0,9537	ns	0,6099	ns	0,1836
1-Decanol	0,7472	ns	0,5535	ns	0,0874	ns	0,388
1-Hexanol	0,3671	ns	0,7567	ns	0,0142	*	0,4876
2-Phenylethanol	0,7041	ns	0,0055	**	0,1584	ns	0,5817
Ethyl Isobutanoate	0,6722	ns	0,0121	*	0,9011	ns	0,4758
Ethyl Butanoate	0,3948	ns	0,1482	ns	0,4455	ns	0,3803
Ethyl 2-Methylbutanoate	0,8055	ns	0,4041	ns	0,1277	ns	0,3833
Ethyl Isovalerate	0,8426	ns	0,0196	*	0,4504	ns	0,4857
Isoamyl Acetate	0,142	ns	0,6976	ns	0,1771	ns	0,3668
Ethyl Hexanoate	0,5368	ns	0,2829	ns	0,1096	ns	0,4205
Hexyl Acetate	0,2297	ns	0,0183	*	0,0288	*	0,6026
Ethyl Heptanoate	0,273	ns	0,8253	ns	0,2574	ns	0,2988
Diethyl Succinate	0,0503	ns	0,0473	*	0,4008	ns	0,5032
Ethyl Octanoate	0,7503	ns	0,7479	ns	0,0195	*	0,4604
Phenethyl Acetate	0,6497	ns	0,7261	ns	0,1659	ns	0,3225
Ethyl Nonanoate	0,1097	ns	< 0.001	***	0,8495	ns	0,6903
Ethyl Decanoate	0,5567	ns	0,1923	ns	0,0339	*	0,5002
Linalool Oxide	0,1038	ns	0,0068	**	0,0244	*	0,6453
Limonene	0,4348	ns	0,4136	ns	0,4108	ns	0,3089
α-Pinene	n.d.		n.d.		n.d.		n.d.
Eucalyptol	n.d.		n.d.		n.d.		n.d.
1,4-Cineole	n.d.		n.d.		n.d.		n.d.
β-Linalool	0,0023	**	0,7969	ns	0,0654	ns	0,5484
α-Ionone	n.d.		n.d.		n.d.		n.d.
α-Terpineol	n.d.		n.d.		n.d.		n.d.
β-Ionone	n.d.		n.d.		n.d.		n.d.
TDN	0,2546	ns	0,9288	ns	0,5341	ns	0,2144
Benzaldehyde	0,1204	ns	< 0.001	***	0,5569	ns	0,7316
Nonanal	n.d.		n.d.		n.d.		n.d.
Octanal	0,5258	ns	0,2651	ns	0,1175	ns	0,4206
Phenylacetaldehyde	0,1984	ns	0,0016	**	0,4021	ns	0,616
2-Heptanone	n.d.		n.d.		n.d.		n.d.
2-Nonanone	0,3001	ns	< 0.001	***	0,2926	ns	0,7392
2-Undecanone	n.d.		n.d.		n.d.		n.d.
β-Damascenone	0,8701	ns	0,7919	ns	0,7771	ns	0,1481
2,4-Di-tert-butylphenol	n.d.		n.d.		n.d.		n.d.

Table S3. Pearson correlation coefficients between selected amino acid–volatile compound pairs involved in the Ehrlich pathway.

Amino acid	Volatile compound	Pearson correlation	
		r	p-value
LEU	Isoamyl Alcohol	0,019	0,9594
ILE	Isoamyl Alcohol	0,177	0,6239
VAL	Isoamyl Alcohol	0,513	0,1297
LEU	Isoamyl Acetate	-0,166	0,6476
PHE	2-Phenylethanol	0,332	0,3484
PHE	Phenethyl Acetate	0,056	0,8773
PHE	Phenylacetaldehyde	-0,327	0,3562
LEU	Ethyl Isovalerate	0,025	0,9462
ILE	Ethyl 2-Methylbutanoate	0,225	0,5312
VAL	Ethyl Isobutanoate	0,606	0,0631

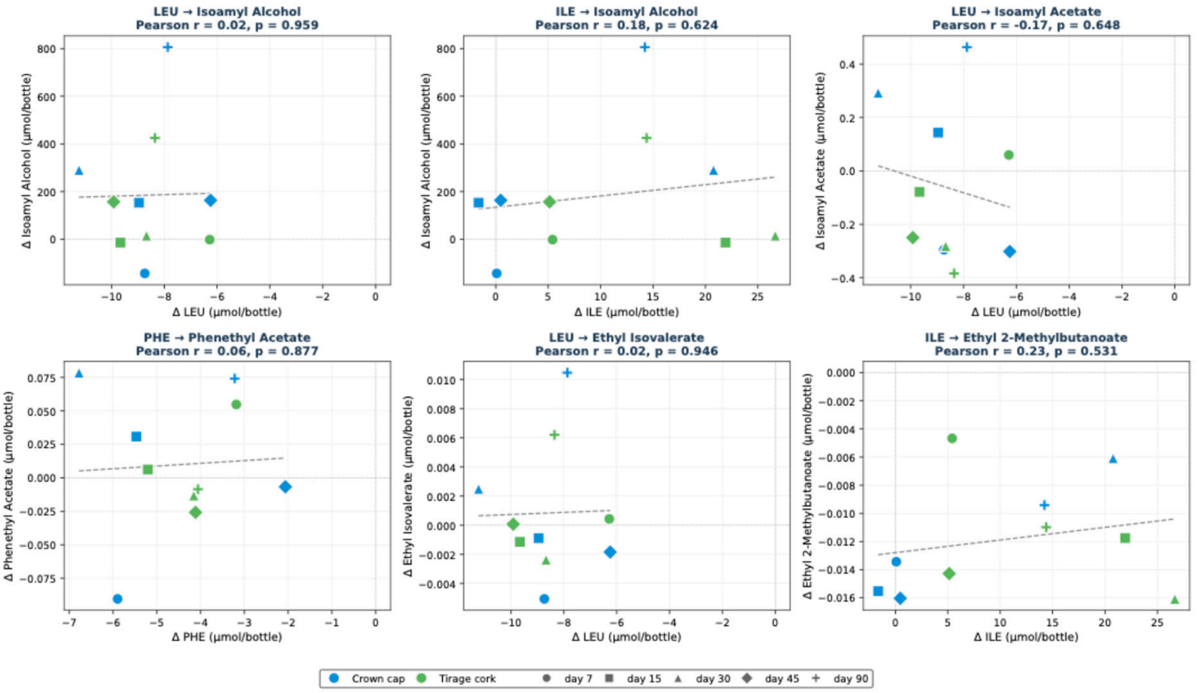

Figure S3. Pearson correlations between amino acid–volatile compound pairs associated with the Ehrlich pathway exhibiting weak associations (|r| < 0.3). Scatter plots show the relationship between variation relative to day 0 (Δ) for the remaining precursor–product pairs not included in Figure 7. Symbols indicate closure type and sampling time.
